# Association of a Genetic Variation in a miR-191 Binding Site in *MDM4* with Risk of Esophageal Squamous Cell Carcinoma

**DOI:** 10.1371/journal.pone.0064331

**Published:** 2013-05-28

**Authors:** Liqing Zhou, Xiaojiao Zhang, Ziqiang Li, Changchun Zhou, Meng Li, Xiaohu Tang, Chao Lu, Helou Li, Qipeng Yuan, Ming Yang

**Affiliations:** 1 Department of Radiation Oncology, Huaian No. 2 Hospital, Huaian, Jiangsu Province, China; 2 College of Life Science and Technology, Beijing University of Chemical Technology, Beijing, China; 3 Hepatobiliary Surgery Department of Qianfoshan Hospital, Shandong University, Jinan, Shandong Province, China; 4 Clinical Laboratory, Shandong Cancer Hospital, Shandong Academy of Medical Sciences, Jinan, Shandong Province, China; 5 Clinical Laboratory, Affiliated Hospital of Taishan Medical University, Taian, Shandong Province, China; Sapporo Medical University, Japan

## Abstract

As an oncoprotein, MDM4 plays a key part in P53 tumor suppressor pathway through negatively regulating P53 function. It has been reported that an rs4245739 A>C polymorphism locating in the *MDM4* 3′-untranslated region creates a miR-191 target site and results in decreased MDM4 expression. Therefore, we investigated the association between this polymorphism and esophageal squamous cell carcinoma (ESCC) risk as well as its biological function *in vivo*. Genotypes were determined in two independent case-control sets consisted of 1128 ESCC cases and 1150 controls from two regions of China. Odds ratios (ORs) and 95% confidence intervals (CIs) were estimated by logistic regression. The impact of the polymorphism on *MDM4* expression was examined with esophagus tissues. Our results demonstrated that the *MDM4* rs4245739 AC and CC genotypes were significantly associated with decreased ESCC risk compared with the AA genotype in both case-control sets (Jinan set: OR = 0.54, 95% CI = 0.35–0.82, *P* = 0.004; Huaian set: OR = 0.68, 95% CI = 0.45–0.99, *P* = 0.049). Stratified analyses revealed that a multiplicative interaction between rs4245739 and smoking or drinking was evident (Gene-smoking: *P*_interactioin_ = 0.022; gene-drinking: *P*_interactioin_ = 0.032). After detecting *In vivo MDM4* mRNA expression, we found that the rs4245739 AC and CC genotype carriers had significantly decreased *MDM4* expression in normal esophagus tissues compared with AA genotype carriers, indicating a consistent genotype-phenotype correlation. Our results elucidate that the *MDM4* rs4245739 polymorphism contributes to susceptibility of ESCC and support the hypothesis that genetic variants, interrupting miRNA-mediated gene regulation, may modify cancer risk.

## Introduction

Esophageal squamous cell carcinoma (ESCC) is one of the most common and fatal cancers in the word, showing a relatively high morbidity in Asian, especially in China [1]. Accumulated epidemiological evidences indicate that tobacco smoking, heavy alcohol drinking, micronutrient deficiency as well as dietary carcinogen exposure might be main environmental risk-factors of this malignant disease [2], [3]. Recent progresses on genome-wide association studies (GWAS) underscore the direct genetic contribution of single nucleotide polymorphisms (SNPs) to ESCC risk, as well as the genetic contribution to ESCC through interaction with the abovementioned environmental causes [4]–[9]. Most SNPs identified so far explain only a small proportion of ESCC genetic basis, leading many to question how the remaining, ‘missing’ heritability can be explained [10], [11]. Therefore, discovery of novel biologically functional and ESCC-risk-associated SNPs might be a potentially valuable path towards illuminating ESCC genetics thoroughly.

As a tumor suppressor, P53 plays a pivotal role in maintaining genomic stability and controlling cell growth as well as apoptosis [12], [13]. MDM2, a key regulator of P53 tumor suppressor pathway, can directly bind to P53 protein, inhibit its activity and lead to its degradation via the ubiquitination pathway [14], [15]. As the structurally homologous protein of MDM2, MDM4 is also a negative regulator of P53 and cooperates with MDM2 to inhibit P53 activity in cellular response to DNA damage [16], [17]. MDM4 can also interact with MDM2 protein via the RING finger domain, and inhibits MDM2 protein degradation [17], [18]. In addition, spontaneous tumorigenesis in transgenetic mice overexpressing MDM4 did show that *MDM4* is a bona fide oncogene *in vivo* cooperating with P53 [19].

In a previous study, Wynendaele et al showed that an SNP (rs4245739 A>C) in the 3′-untranslated region (3′-UTR) of *MDM4* that creates a putative target site for miR-191 [20]. This group and others also showed that miR-191 could selectively bind to MDM4-C allele mRNA but not MDM4-A allele mRNA, which resulting in a statistically significant increased expression of *MDM4* mRNA and protein levels among *MDM4* rs4245739 A allele carriers in ovarian cancer and retinoblastoma [20], [21]. Moreover, ovarian cancer patients with rs4245739 AA genotype who do not express the estrogen receptor had a 4.2-fold [95% confidence interval (CI) = 1.2–13.5; *P* = 0.02] increased risk of recurrence and 5.5-fold (95% CI = 1.5–20.5; *P* = 0.01) increased risk of tumor-related death compared with cases with AC or CC genotype [20]. Due to the causative link between high levels of MDM4 and tumorigenesis, we hypothesized that the *MDM4* rs4245739 SNP may be also involved in ESCC development through impacting miR-191-mediated differential regulation of MDM4 expression. To test this hypothesis, we conducted a large case–control study of ESCC from two different regions of China. To validate the biological function of this SNP *in vivo*, we examined the association between its genotypes and *MDM4* mRNA expression levels in normal and cancerous esophagus tissues.

## Materials and Methods

### Study Subjects

There were two case-control sets in the current study. (a) Jinan case-control set: 540 patients with ESCC from Shandong Cancer Hospital, Shandong Academy of Medical Sciences (Jinan, Shandong Province, China) and sex- and age-matched (±5 years) 550 controls. Patients were recruited between June 2009 and April 2012 at Shandong Cancer Hospital. Control subjects were randomly selected from a pool of 4500 individuals from a community cancer-screening program for early detection of cancer conducted in Jinan city during the same time period as the patients were collected. (b) Huaian case-control set: 588 ESCC patients from Huaian No. 2 Hospital (Huaian, Jiangsu Province, China) and sex- and age-matched 600 controls. Patients were consecutively recruited between January 2009 and February 2012 at Huaian No. 2 Hospital. Controls were cancer-free individuals selected from a community cancer-screening program (3600 individuals) for early detection of cancer conducted in Huaian city during the same time period as the patients were collected. The diagnosis of all patients was histologically confirmed. Individuals who smoked one cigarette per day for over 1 year were considered as smokers. Subjects were considered as alcohol drinkers, if they drank at least once per week. Twenty-nine esophagus normal tissues adjacent to the tumors and twenty nine paired ESCC tissues were obtained from surgically removed specimens of patients in Huaian No. 2 Hospital. The normal tissues sampled at least 2 cm away from the margin of the tumor. Part of the two case-control sets and the tissue samples has been reported previously [22], [23]. All subjects were ethnic Han Chinese. This study was approved by the Institutional Review Boards of Huaian No. 2 Hospital and Shandong Cancer Hospital, Shandong Academy of Medical Sciences. At recruitment, written informed consent was obtained from each subject.

### SNP Genotyping

*MDM4* rs4245739 A>C genotypes were determined using PCR-based restriction fragment length polymorphism (RFLP). During genotyping, the primers used for amplifying DNA segments with the SNP site (the mismatch base is underlined) were 5′-AAGACTAAAGAAGGCTGGGG-3′ and 5′-TTCAAATAATGTGGTAAGTGACC-3′. The PCR was performed with a 25 µL reaction mixture containing 100 ng of DNA, 0.1 mmol/L of each primer, 0.2 mmol/L of deoxynucleoside triphosphate, 1.0 U of rTaq DNA polymerase (TaKaRa), 1×reaction buffer, and 1.5 mmol/L MgCl_2_. The PCR profile consisted of an initial melting step of 2 minutes at 95°C, followed by 35 cycles of 30 seconds at 94°C, 30 seconds at 58°C, 30 seconds at 72°C, and a final elongation step of 10 minutes at 72°C. Restriction enzyme *Msp*I (New England Biolabs) was utilized to distinguish the rs4245739 A>C genotypes. A 15% random sample was reciprocally tested by different person, and the reproducibility was 99.9%. In addition, a 5% random sample was also examined by Sanger sequencing, and the reproducibility was 100%.

### Real-time Analysis of MDM4 mRNA

SYBR-Green real-time quantity PCR method was used to examine *MDM4* mRNA levels in normal and cancerous esophagus tissues as described previously [22], [24], [25]. In brief, total RNA was isolated and converted to cDNA using the ReverTra Ace qPCR RT Kit (TOYOBO). Relative gene expression quantitation for *MDM4* and *GAPDH* as an internal reference gene was carried out using the ABI 7500 real-time PCR system in triplicates. The primers used for *MDM4* were 5′-CTACCGAGTGTCTGTCTAAG-3′ and 5′-TCCTGGGTGTTTGTATTT-3′; and for *GAPDH* were 5′-AACAGCGACACCCATCCTC-3′ and 5′-CATACCAGGAAATGAGCTTGACAA-3′.

### Statistics

Pearson’s χ^2^ test was used to examine the differences in demographic variables and genotype distributions of *MDM4* rs4245739 polymorphism between ESCC patients and controls. Associations between *MDM4* rs4245739 genotypes and risk of the development of ESCC were estimated by OR and their 95% CIs computed using unconditional logistic regression model. All ORs were adjusted for age and sex, where it was appropriate. We tested the null hypotheses of multiplicative gene-covariate interaction and evaluated departures from multiplicative interaction models by including main effect variables and their product terms in the logistic regression model [26]–[28]. A *P* value of less than 0.05 was used as the criterion of statistical significance, and all statistical tests were two-sided. All analyses were performed with SPSS software package (Version 16.0, SPSS Inc., Chicago, IL).

## Results

We observed no statistically significant differences between cases and controls for Jinan case-control set and Huaian case-control set in terms of median age and sex distribution (all *P*>0.05), indicating that the frequency matching was adequate (Table 1). However, there were more smokers among ESCC patients compared with control subjects in both case-control sets (Jinan set: 65.5% vs. 51.8%, *P*<0.001; Huaian set: 74.3% vs. 33.8%, *P*<0.001). In addition, more alcohol drinkers among ESCC cases were observed than among controls in these two sets (Jinan set 55.6% vs. 45.6%, *P* = 0.001; Huaian set: 56.8% vs. 40.3%, *P*<0.001).

**Table 1:** Distribution of selected characteristics among ESCC cases and controls.

Variable	Jinan case-control set			Huaian case-control set		
	Patients	Controls	*P*-value a	Patients	Controls	*P*-value^a^
	*n* (%)	*n* (%)		*n* (%)	*n* (%)	
	540	550		588	600	
Age (year)^b^			0.167			0.725
≤56(or 59)	271(50.2)	299(54.4)		288(49.0)	300(50.0)	
>56(or 59)	269(49.8)	251(45.6)		300(51.0)	300(50.0)	
Sex			0.193			0.678
Male	428(79.3)	453(82.4)		413(70.2)	428(71.3)	
Female	112(20.7)	97(17.6)		175(29.8)	172(28.7)	
Smoking status			<0.001			<0.001
No	186(34.4)	265(48.2)		151(25.7)	397(66.2)	
Yes	354(65.5)	285(51.8)		437(74.3)	203(33.8)	
Drinking status			0.001			<0.001
No	240(44.4)	299(54.4)		254(43.2)	358 (59.7)	
Yes	300(55.6)	251(45.6)		334(56.8)	242(40.3)	

Note: ESCC, esophageal squamous cell carcinoma.

a Two-sided χ^2^ test.

b Median ages of patients for Jinan set and Huaian set are 56 and 59 years.

The allelic and genotype frequencies of *MDM4* rs4245739 A>C polymorphism are summarized in Table 2. The frequency for the rs4245739 C allele was 0.067 and 0.077 among healthy controls from Jinan set and Huaian set, and 0.038 and 0.053 among ESCC patients from Jinan set and Huaian set. All observed genotype frequencies in both controls and patients conform to Hardy-Weinberg equilibrium. Distributions of these *MDM4* genotypes were then compared among ESCC cases and controls. The frequencies of *MDM4* rs4245739 AA and AC or CC genotypes among cases were significantly different from those among controls in Jinan set (χ^2^ = 10.26, *P* = 0.004, *df* = 1) (Table 2). Similarly, the frequencies of *MDM4* rs4245739 AA and AC or CC genotypes among cases were significantly different from those among controls in Jinan set (χ^2^ = 6.68, *P* = 0.049, *df* = 1) (Table 2).

**Table 2:** Genotype frequencies of *MDM4* rs4245739 polymorphism among patients and controls and their association with ESCC risk.

Genotypes		*MDM4* rs4245739 A>C			
		Patients *n*(%)	Controls *n* (%)	OR^a^ (95% CI)	*P*-value
Jinan set		*n* = 540	*n* = 550		
	AA	501(92.8)	478(86.9)	Reference	
	AC	37(6.9)	70(12.7)	0.52(0.34–0.80)	0.003
	CC	2(0.3)	2(0.4)	NC	NC
	AC+CC	39(7.2)	72(13.1)	0.54(0.35–0.82)	0.004
	*P*_trend_ b			0.002	
Huaian set		*n* = 588	*n* = 600		
	AA	529(90.0)	510(85.0)	Reference	
	AC	56(9.5)	88(14.7)	0.66(0.45–0.98)	0.047
	CC	3(0.5)	2(0.3)	NC	NC
	AC+CC	59(10.0)	90(15.0)	0.68(0.45–0.99)	0.049
	*P*_trend_ b			0.018	
Total		*n* = 1128	*n* = 1150		
	AA	1030(91.3)	988(85.9)	Reference	
	AC	93(8.2)	158(13.7)	0.63(0.47–0.83)	0.001
	CC	5(0.5)	4(0.4)	NC	NC
	AC+CC	98(8.7)	162(14.1)	0.65(0.49–0.85)	0.002
	*P*_trend_ b			1.6×10^−4^	

Note: ESCC, esophageal squamous cell carcinoma; NC, not calculated; OR, odds ratio; CI, confidence interval.

a Data were calculated by logistic regression with adjustment for age, sex, smoking and drinking status.

b Test for trend of odds was two-sided and based on likelihood ratio test assuming a multiplicative model.

Unconditional logistic regression analyses were used to calculate associations between genotypes of *MDM4* rs4245739 A>C SNP and ESCC risk (Table 2). The *MDM4* rs4245739 C allele was shown to be a protective allele. Individuals having the rs4245739 AC genotype had an OR of 0.52 (95% CI = 0.34–0.80, *P* = 0.003) or 0.66 (95% CI = 0.45–0.98, *P* = 0.047) for developing ESCC in Jinan Set or Huaian set, respectively, compared with individual having the rs4245739 AA genotype. In Jinan set, the rs4245739 AC and CC genotypes had a 0.54-fold decreased risk for ESCC compared with the rs4245739 AA genotype (95% CI = 0.35–0.82, *P = *0.004). Similarly, logistic regression analyses revealed that individuals with the rs4245739 AC and CC genotypes were also significantly associated with decreased ESCC risk in Huaian set (OR = 0.68, 95% CI = 0.45–0.99, *P = *0.049) (Table 2). In the pooled analyses, we found that carriers of the rs4245739 AC and CC genotypes had a 0.65-fold decreased risk to develop ESCC compared to the AA genotype carriers (95% CI = 0.49–0.85, *P* = 0.002) (Trend test, *P* = 1.6×10^−4^) (Table 2). All ORs were adjusted for sex, age, smoking and alcohol drinking status.

The risk of ESCC associated with the *MDM4* rs4245739 genotypes was further examined by stratifying for age, sex, smoking and alcohol drinking status using the combined data of two Chinese case-control sets (Table 3). In stratified analyses with age, rs4245739 AC and CC genotypes were significantly associated with decreased risk in both subjects aged 57 years or younger (OR = 0.62, 95% CI = 0.41–0.94, *P* = 0.023) and subjects aged older than 57 years (OR = 0.64, 95% CI = 0.44–0.94, *P* = 0.021). No significant gene-age interaction was observed (*P*_interaction_ = 0.796). Compared with the *MDM4* rs4245739 AA genotype, a significantly decreased risk of ESCC was associated with AC and CC genotypes only among females (OR = 0.46, 95% CI = 0.26–0.80, *P* = 0.006), but not among males (OR = 0.74, 95% CI = 0.53–1.03, *P* = 0.074). There was a marginally significant gene-sex interaction (*P*_interaction_ = 0.080).

**Table 3:** Association between *MDM4* rs4245739 A>C variant and ESCC risk stratified by selected variables.

Variable	*MDM4* rs4245739 A>C				*P*_interaction_ c
	AA^a^	AC+CC^a^	OR^b^ (95% CI)	*P*-value	
Age (year)					0.796
≤57	513/522	46/77	0.62(0.41–0.94)	0.023	
>57	517/466	52/85	0.64(0.44–0.94)	0.021	
Sex					0.080
Male	770/780	71/101	0.74(0.53–1.03)	0.074	
Female	260/208	27/61	0.46(0.26–0.80)	0.006	
Smoking status					0.022
Nonsmoker	305/544	32/118	0.51(0.33–0.77)	0.002	
Smoker	725/444	66/44	0.91(0.60–1.37)	0.654	
Alcohol drinking					0.032
No	450/538	44/119	0.52(0.35–0.76)	0.001	
Yes	580/450	54/43	0.95(0.61–1.46)	0.799	

Note: ESCC, esophageal squamous cell carcinoma; OR, odds ratio; CI, confidence interval.

a Number of case patients with genotype/number of control subjects with genotype.

b Data were calculated by logistic regression, adjusted for sex, age, smoking, and drinking status, where it was appropriate.

c *P* values for gene-environment interaction were calculated using the multiplicative interaction term in SPSS software.

Because tobacco smoking and alcohol drinking are predisposing factors for ESCC, we then investigated whether a gene–environment interaction existed between the *MDM4* rs4245739 polymorphism and these risk factors (Table 3). In nonsmokers, compared with the rs4245739 AA carriers, individuals with AC and CC genotypes had a 0.51-fold decreased risk to develop ESCC (95% CI = 0.33–0.77, *P* = 0.002).There was no significantly decreased risk (OR = 0.91, 95% CI = 0.60–1.37, *P* = 0.654) for smokers with AC and CC genotypes compared with AA smokers. A multiplicative gene–smoking interaction was also found with *P*_interactioin_ equaling to 0.022. Nondrinkers carrying rs4245739 AC and CC genotypes showed significantly decreased risk to develop ESCC compared with AA carriers who did not drink (OR = 0.52, 95% CI = 0.35–0.76, *P* = 0.001). However, no association between rs4245739 AC and CC genotypes and ESCC risk was observed in drinkers (OR = 0.95, 95% CI = 0.61–1.46, *P* = 0.799) (Table 3). An evident gene-drinking interaction exists (*P*_interactioin_ = 0.032).

**Figure 1:**
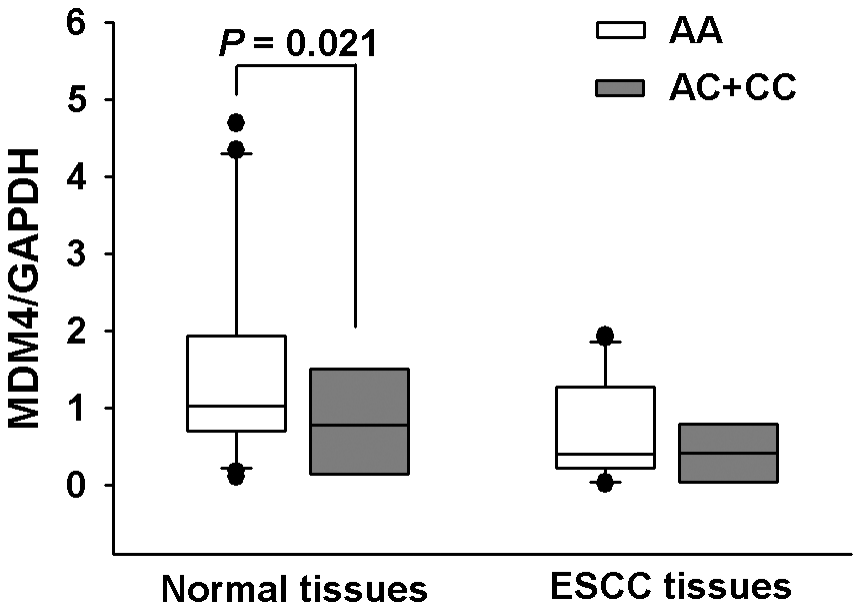
*MDM4* mRNA expression in normal and cancerous esophagus tissues grouped by *MDM4* rs4245739 A>C genotypes. Individuals with the rs4245739 AC and CC genotypes had significantly lower *MDM4* mRNA levels (mean ± SE) than those with AA genotype in normal esophagus tissues (1.507±0.260 [n = 25] vs. 0.808±0.356 [n = 4], *P = *0.021), but not in ESCC tissues (0.737±0.139 [n = 25] vs. 0.720±0.210 [n = 4], *P*>0.05).

Because rs4245739 C-to-A change could destroy RNA::RNA interaction between miR-191 and *MDM4* mRNA and increased *MDM4* expression in cancer cells, we studied if there is an allele-specific effect of rs4245739 SNP on *MDM4* expression in esophagus tissues. As shown in Figure 1, individuals with the rs4245739 AC and CC genotypes had significantly lower *MDM4* mRNA levels (mean ± SE) than those with AA genotype in normal esophagus tissues (1.507±0.260 [n = 25] vs. 0.808±0.356 [n = 4], *P = *0.021), but not in ESCC tissues (0.737±0.139 [n = 25] vs. 0.720±0.210 [n = 4], *P*>0.05).

## Discussion

In this study, we employed a hypothesis-driven approach to examine the association between a *MDM4* functional SNP and risk of developing ESCC in a case-control design. To the best of our knowledge, this is the first case-control study to investigate the association between the *MDM4* rs4245739 polymorphism and ESCC risk. We found significantly decreased ESCC risks among carriers of *MDM4* rs4245739 C allele compared with those with A allele in Chinese. In the genotype-phenotype correlation analysis of 29 human ESCC and paired esophagus tissue samples, rs4245739 AC and CC genotypes were associated with a statistically significant decrease of *MDM4* mRNA expression. These results are consistent to functional relevance of rs4245739 polymorphism in miR-191-mediaed regulation of MDM4 expression in malignant transformation of human cells [20], [21]. Our observations also support the hypothesis that genetic variants in miRNA illegitimate target sites of tumor suppressor genes or oncogenes may influence cancer susceptibility.

Some functional naturally occurring genetic variants in genes of the P53 tumor suppressor pathway (i.e. *P53* Arg72Pro and MDM2 T309G) have been identified and associated with ESCC risk [29]–[32]. These results highlight the importance of genes in the P53 tumor suppressor pathway during ESCC development. Since accumulated evidences supporting a key role for MDM4 in the regulation of P53 tumor suppression function [14]–[17], it is biologically plausible to speculate that the decreased ESCC risk observed among *MDM4* rs4245739 C allele carriers result from the increased tumor suppressor activity of P53.

Both tobacco smoking and alcohol are well-known environmental causes of DNA damage [33], [34]. As a key DNA damage response protein, inhibition of P53 by MDM2 and MDM4 can result in delayed DNA repair, increased genome instability and tumorigenesis. Therefore, down-regulated expression of MDM4 in individuals carrying rs4245739 C allele can lead to elevated DNA repair ability mediated by P53, and, thus, decreased cancer risk. However, a large amount of exposure to tobacco smoking and alcohol may dismiss the difference arising from the *MDM4* rs4245739 polymorphism. This could explain, at least in part, the gene-smoking and gene-drinking interaction in the current study.

In all, our results demonstrated that functional *MDM4* rs4245739 SNP was associated with a significantly decreased risk of ESCC in Chinese populations. The associations between SNPs and ESCC risk are especially noteworthy in individuals who did not smoke or drink. Additionally, these results may support the hypothesis that genetic variants can interrupt miRNA-mediated gene regulation and this kind of regulatory SNPs might be important modifiers of ESCC risk.
